# Spore sensitivity to sunlight and freezing can restrict dispersal in wood-decay fungi

**DOI:** 10.1002/ece3.1589

**Published:** 2015-07-22

**Authors:** Veera Norros, Elina Karhu, Jenni Nordén, Anssi V Vähätalo, Otso Ovaskainen

**Affiliations:** 1 Department of Biosciences, Metapopulation Research Centre, University of HelsinkiP.O. Box 65, FI-00014, Helsinki, Finland; 2 Marine Research Centre, Finnish Environment InstituteP.O. Box 140, FI-00251, Helsinki, Finland; 3 Natural History Museum, University of OsloP.O. Box 1172 Blindern, NO-0318, Oslo, Norway; 4 Section for Genetics and Evolutionary Biology, Department of Biosciences, University of OsloP.O. Box 1066 Blindern, NO-0316, Oslo, Norway; 5 Department of Environmental Sciences, University of HelsinkiP.O. Box 65, FI-00014, Helsinki, Finland

**Keywords:** Basidiomycetes, connectivity, germination, habitat fragmentation, life-history evolution, long-distance dispersal, mortality, movement, spore viability, stress tolerance, ultraviolet radiation

## Abstract

Assessment of the costs and benefits of dispersal is central to understanding species' life-history strategies as well as explaining and predicting spatial population dynamics in the changing world. While mortality during active movement has received much attention, few have studied the costs of passive movement such as the airborne transport of fungal spores. Here, we examine the potential of extreme environmental conditions to cause dispersal mortality in wood-decay fungi. These fungi play a key role as decomposers and habitat creators in forest ecosystems and the populations of many species have declined due to habitat loss and fragmentation. We measured the effect of simulated solar radiation (including ultraviolet A and B) and freezing at −25°C on the spore germinability of 17 species. Both treatments but especially sunlight markedly reduced spore germinability in most species, and species with thin-walled spores were particularly light sensitive. Extrapolating the species' laboratory responses to natural irradiance conditions, we predict that sunlight is a relevant source of dispersal mortality at least at larger spatial scales. In addition, we found a positive effect of spore size on spore germinability, suggesting a trade-off between dispersal distance and establishment. We conclude that freezing and particularly sunlight can be important sources of dispersal mortality in wood-decay fungi which can make it difficult for some species to colonize isolated habitat patches and habitat edges.

## Introduction

Dispersal is necessary for the long-term persistence of lineages of organisms, but it also has costs. Current ecological literature emphasizes an individual-based approach to movement and dispersal, viewing them as life-history processes determined by the interplay of the traits of the organism and its external environment and subjected to evolutionary selection pressures and constraints (Nathan et al. 2008; Bonte et al. 2012). From the individual perspective, the costs of dispersal can be measured in terms of energy, time, mortality risk, and lost opportunities (Bonte et al. 2012). Costs of dispersal can result in trade-offs between dispersal and other life-history traits, or between different stages of the dispersal process (Bonte et al. 2012). A correct evaluation of the costs of dispersal is crucial in order to estimate the connectivity of landscapes from the point of view of organisms and further to understand and predict where species occur now and in the future (Kokko and Lopez-Sepulcre 2006; Burgess et al. 2012).

In passive dispersers with airborne propagules, dispersal costs include mortality at departure due to propagule predation, loss of viability caused by harsh atmospheric conditions during transfer, and mortality or loss of opportunity at settlement as some propagules end up in unsuitable habitats and microsites. In addition, there are likely to be substantial predeparture energy costs for developing morphological structures related to dispersal (Bonte et al. 2012). In sessile species, structures such as the elaborate fruit bodies and thick spore walls of many fungi can be considered to contribute to the predeparture dispersal costs, although these structures also play a role in reproduction and establishment. In passive dispersers, most study effort has concentrated on costs at departure and settlement (Bonte et al. 2012). However, costs during the transfer phase can also be substantial, for instance, in species with small propagules that can remain in the atmosphere for a long time (Wilkinson et al. 2012). Based on mathematical considerations, Reynolds (2013) suggested that the strategy of many passive dispersers may be to aim at the shortest unique dispersal distance – far enough to get away from conspecific competitors but not too far for the transfer costs to rise too high.

Most fungi disperse by microscopic spores carried primarily by the wind or water (Deacon 1997). In the multicellular members of the phylum Basidiomycota, sexual reproduction occurs when a dikaryotic individual forms specialized fruiting structures in which meiosis occurs and haploid spores (called basidiospores) are released. Upon landing on suitable substrate, a haploid spore germinates and develops into monokaryotic mycelium which can fuse with another monokaryotic mycelium or a spore of a compatible mating type to form a dikaryotic individual. In basidiomycetes, sexual spores are the dominant way of reproduction although some species reproduce additionally or exclusively by asexual spores. Fragmentation of mycelium is a form of vegetative reproduction that is of less importance for unit-restricted fungi (such as wood-inhabiting fungi) than for non-unit-restricted fungi (such as soil-inhabiting fungi) (Deacon 1997). Compared to animals and plants, dispersal mortality in fungi with airborne spores has been studied very little and almost exclusively in plant pathogenic species. Based on existing knowledge, solar radiation, extreme temperatures, and desiccation are considered the most important causes of spore mortality during dispersal (Buller 1909; Gregory 1973; Rotem and Aust 1991; Parnell et al. 1998; Mitakakis et al. 2003; Ghajar et al. 2006; Isard et al. 2006; Kanetis et al. 2010). Among fungi, wood-decay basidiomycetes are characterized by typically very small, elongated, thin-walled, and colorless basidiospores that have a very high dispersal potential but do not appear well-adapted to harsh conditions (Kramer 1982; Parmasto and Parmasto 1992; Wilkinson et al. 2012; Norros et al. 2014). However, the sensitivity of the spores to environmental conditions has been confirmed in controlled studies for only a very few wood-decay species (although see Buller 1909).

Dispersal mortality could be especially severe in wood-decay fungi that occupy a highly fragmented forest landscape in which distances between habitat patches are long. All of the expected causes of mortality (solar radiation, extreme temperatures, and desiccation) can be expected to be more severe when spores are dispersed across wide open areas instead of within a continuous forest. Moreover, the microclimate that awaits spores landing on their substrate can be less benign due to the increased proportion of habitat edges, resulting in higher mortality at settlement (Saunders et al. 1991).

In this study, we assess whether solar radiation and freezing can be significant sources of dispersal mortality in wood-decay fungi occurring in the fragmented forest landscapes of northern Europe. In Finland, Sweden, and Norway, intensive forestry has resulted in dramatically decreased density of the dead wood substrates required by wood-decay fungi and loss of habitat connectivity at the landscape scale (Kouki et al. 2001; Siitonen 2001; Jonsson et al. 2005). As a result, many wood-decay species have declined. In the best-known group, the polypores (poroid Aphyllophorales) currently more than 40% of the species in Finland and Norway are nationally red-listed (Brandrud et al. 2010; Kotiranta et al. 2010). Evidence is accumulating that the declining species are rare substrate specialists that require high habitat or even substrate connectivity (Edman et al. 2004b; Berglund and Jonsson 2008; Jönsson et al. 2008; Olsson et al. 2011; Nordén et al. 2013), and many of them are likely to be limited by dispersal despite the high dispersal potential of their spores (Norros et al. 2012). Further, the occurrence probability of some wood-decay fungi has been found to be decreased at habitat edges, probably due to the more adverse physical conditions (Snäll and Jonsson 2001; Siitonen et al. 2005).

Our study is based on laboratory experiments in which we exposed basidiospores collected from naturally occurring fruit bodies of wood-decay fungi to 4–48 h of simulated solar radiation (including ultraviolet A and B but henceforth called light for simplicity) or freezing (−25°C), simulating extreme physical conditions during dispersal. We examined the germinability of spores, defined as the proportion of spores germinating after 24 h of culturing. More specifically, we looked at three response variables: the initial germinability of spores (*g*_0_), that is, germinability of untreated spores, and the resistance of spores to light (*R*_L_) and freezing (*R*_F_), defined as the germinability after exposure divided by the germinability in a control treatment (darkness in 25°C). Using the observed decay of spore germinability in the light treatments, we estimated dispersal mortality caused by natural sunlight. Finally, we analyzed whether species-specific spore morphology is related to initial spore germinability (*g*_0_), light (*R*_L_), and freezing resistance (*R*_F_) in wood-decay fungi.

## Material and Methods

### Spore collection

Spores for our laboratory experiments were collected in April–May and August–October 2008 at two sites in southern Finland. The site Lammi (61.053° N, 25.045° E) and the site Evo (61.238° N, 25.059° E) are separated by ca. 20 km. These two sites were chosen for their species-rich but contrasting wood decayer communities (respectively dominated by deciduous vs. conifer wood associated species) in order to maximize the pool of potential study species. For each study species, we collected five spore samples, each of these from an individual fruit body growing on a separate log; thus, the five samples represented five fungal individuals. The spores of 1–2 species were collected on the first and/or second day of the week and processed in the laboratory during the rest of the week. All samples of the 1–2 species handled in a given week were put through the treatments simultaneously. Each species was collected and studied during 1 week only, and the samples of each species were always collected from the same site on the same day.

The labor-intensive laboratory work limited the number of species studied. The studied species were selected among those polypore species for which a sufficient number of actively sporulating individuals were found. Our aim was to select polypore species representing the range of different spore sizes and shapes, in terms of spore volume (*V*) and elongation (*e*), and including both species with thin- and thick-walled spores (wall thickness *w*). We selected these variables to describe the spore morphology as wall thickness as well as average spore length (*L*) and width (*W*) are routinely recorded in taxonomical work, and spore volume and elongation can be calculated from the latter two. Spore volume was calculated as *V* = (*π*/6) *LW*^2^ assuming a prolate ellipsoid spore shape; spore elongation was calculated as *e* = *L*/*W*. The values of *L* and *W* as well as the classification of thick- or thin-walled spores were based on taxonomic literature (Niemelä 2005).

We collected the spore samples on plastic foil pieces that were attached with pins to the spore-releasing surface (hymenophore), one foil piece per fruit body. Spores were allowed to settle on the foil pieces for 14–24 h, always spanning over at least one whole night. The spore-containing foil pieces were taken to the laboratory and the treatments started immediately after collection.

### Treatments and culturing

In the laboratory, a fraction of each spore sample (i.e., spore-containing foil piece) was first taken for culturing, to represent the initial germinability (*g*_0_) of the spores from the sampled individuals. The remaining spores of the sample were divided into three different treatments. The treatments were simulated sunlight at 25°C, dark at −25°C, and dark at 25°C (control) for four different lengths of time (4, 16, 24, and 48 h for *Phellinus igniarius* and *P. punctatus*; 4, 8, 24, and 48 h for the rest of the species), resulting in 12 different treatments altogether. For the second observation time, either 8 or 16 h was chosen so that night work was avoided. The freezing treatment at −25°C was chosen to represent an extreme temperature that spores dispersing in late autumn or early spring may experience in northern Europe, especially if they are transported to high altitudes in the atmosphere – the lapse rate of temperature with altitude is ca. 6°C per km for moist air (Seinfeld and Pandis 2006), and fungal spores have been sampled even at >10 km height in the atmosphere (Gregory 1973). Moreover, there is evidence that species with perennial fruit bodies can sometimes produce spores even during brief thaws in the winter (Nuss 1975), after which the temperature can rapidly drop again. The control temperature 25°C was higher than the outside temperature at the time of spore collection (not monitored but ranging ca. −5°C to +20°C during the sampling time), but was chosen to match the temperature in the light treatment, which was difficult to keep lower due to the heating effect of the sunlight lamps. As there are observations of wood decayer spores remaining viable for at least a week (Edman et al. 2004a) and up to months (Mukhin and Votintseva 2004) at warm and dry indoor conditions, we expected 25°C to be a relatively benign temperature for the spores.

**Figure 1:**
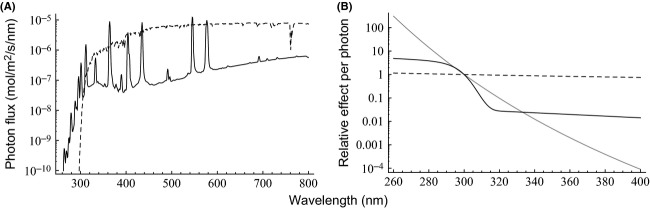
(A) The measured irradiation spectra of the Ultra-Vitalux lamps (solid) and the average solar irradiation spectrum as reported by Chu and Liu (2009) (dashed), scaled to a global radiation of 1000 Wm^−2^ at 300–4000 nm, approximating the global radiation (i.e., including both direct and scattered or reflected radiation) in Finland at noon on a cloudless summer day. (B) The alternative spectral weighing functions used to estimate the biologically effective radiation dose in the treatments shown standardized to 1 at 300 nm. Gray: Quaite et al. (1992) as formulated by Musil (1995). Black, solid: Flint and Caldwell (2003). Black, dashed: the energy content per photon relative to the energy content of a 300 nm photon.

We used two 300 W ultraviolet (UV) light bulbs (Osram Ultra-Vitalux) to simulate sunlight. These lamps emit radiation at the UV-B, UV-A, visible light, and infrared regions (Fig. 1), and are described by the manufacturer as simulating natural sunlight at high altitudes (e.g., mountainous areas). The lamps were hung next to each other, separated by ca. 30 cm, and the spore-containing foil pieces were placed under the lamps on a horizontal plane that was at a distance of 40 cm from the plane of the lamps. The whole setup was placed in a cold room to keep the temperature benign (up to ca. 25°C) despite the heat generated by the lamps. The spores remained on the foil pieces also in the control and freezing treatments. The control treatment was conducted in a culturing cabinet and the freezing treatment in a chest freezer. We monitored the temperature, relative humidity, and light intensity in the treatments with data loggers (Onset HOBO H8). In addition, we later reconstructed the setup and used a spectroradiometer (Macam SR 991) to measure the irradiance spectrum incident on the spore samples.

After the treatments, the spores were cultured for 24 h in darkness at 20–24°C on 9-cm Petri plates filled with 2% malt agar with pH adjusted to 5.5. The proportion of spores that had begun to germinate was determined by scanning the agar surface through the bottom of the closed plate with a compound microscope. We classified spores as germinating if an emerging germ tube could be distinguished. We aimed at counting 100 spores from each plate, but for some individual plates, spore density was so low that only fewer (down to ca. 10) spores could be found. Spore density on a plate was not controlled but as the proximity to other spores can affect germinability (Hobot and Gull 1980; Barrios-Gonzáles et al. 1989; Chitarra et al. 2004), we avoided counting areas with very high spore density.

We calculated three measures of spore viability: initial germinability (*g*_0_; proportion of germinating spores), light resistance (*R*_L_; ratio of germinability in light treatment to germinability in control treatment), and freezing resistance (*R*_F_; ratio of germinability in freezing treatment to germinability in control treatment). We used the 4-h treatments for measuring *R*_L_ and *R*_F_, as most of the decrease in germinability occurred already by this time.

We would like to note here that our choice of fixed 24 h' culturing time (also used, e.g., by Tsuneda and Kennedy (1980) and Edman et al. (2004a)) can be criticized on two accounts. First, although we are not aware of systematic studies on the subject, the experience of experts is that germination time in culture varies among species and can sometimes be several days or even weeks (O. Miettinen, pers. comm.). Second, it is conceivable that the light and freezing treatments could delay germination compared to the control treatment, which in our approach would be interpreted as reduction in germinability. As to the first point, while a longer culturing time would have been more ideal, 24 h was chosen based on pilot experiments as a compromise between allowing as much time as possible for germination on one hand and terminating the culturing before the plates were overgrown by contaminants on the other hand. The presence of contaminants was unavoidable as the spore samples were collected in the field. Given this restriction, we excluded species with very slow (or nonexistent) germination in culture based on our own and colleagues' previous experience already at the spore collection phase. As to the second point, in a subsequent study (J. Nordén, E. Karhu, O. Ovaskainen, A. V. Vähätalo, K.-H. Larsson, M. Edman, V. Norros, unpubl. ms.), we considered and tested adjusting the culturing time according to the observed phase of germination in each sample for three species (*Phellinus ferrugineofuscus*, *Phellinus viticola*, *Fomitopsis pinicola*; the latter two are included also in the present study). For these species, a longer culturing time (up to 92 h) did not change the germinability of treated spores relative to the control. Thus, we consider it likely that the difference between light and freezing treatments and the control largely represents true reduction in germinability; however, the possibility of delayed germination cannot be completely excluded.

### Estimating the effect of light-induced reduction in germinability on dispersal distances

Our light and freezing treatments represent extreme conditions and thus quantify the maximal dispersal mortality that can be expected through these factors. Here, we describe an attempt to translate the experimental reduction in germinability to that occurring in typical dispersal conditions. For freezing, there is no obvious basis for extrapolating the laboratory responses to milder conditions; however, it is reasonable to expect that the physiological effects of freezing are at least qualitatively similar regardless of the exact temperature. For light, on the other hand, we made such extrapolations based on the assumption that the extent of the radiation damage to spores depends on the total radiation dose. This assumption does not necessarily hold but is supported by a fair amount of empirical evidence across different organisms (e.g., McKenzie et al. 2011, but see Ghajar et al. 2006).

To assess how much light-induced reduction in germinability can be expected to affect the dispersal distances of wood-decay fungi in nature, we used the decay of germinability over time in the light treatment to calculate the half-life of spores under simulated sunlight 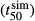. The half-lives and the measured wavelength spectrum of the simulated light as well as that of natural sunlight (Chu and Liu 2009) were then used to predict the decay of spore germinability under realistic scenarios of solar radiation which were based on global radiation values measured across different seasons and latitudes in Finland by the Finnish Meteorological Institute (Anonymous 1993). The global radiation measurements were made in open areas; thus, our scenarios correspond to dispersal above the canopy or across open areas.

**Figure 2:**
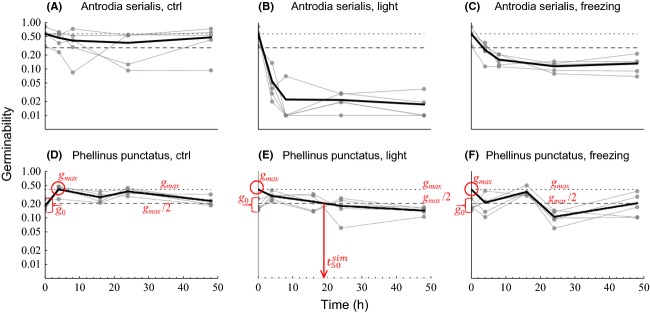
The change in germinability over time (h) in the control (dark at 25°C; A, D), light (simulated sunlight at 25°C; B, E), and freezing (dark at -25°C; C, F) treatments shown here for two example species with contrasting responses (Antrodia serialis, A-C; Phellinus punctatus, D-F) and for all examined species in Appendix S2, Fig. S1. The gray points connected by each thin gray line show the germinability of spores from one sampled individual in the course of the treatment, with the value at time 0 showing the initial germinability *g*_0_ (i.e., germinability without treatment). The thick black line is the mean germinability in each treatment, averaged over the different individuals. The horizontal dotted and dashed lines show, respectively, the maximal (*g*_max_) and 50% of the maximal (*g*_max_/2) mean germinability observed in the control treatment. In the control treatment, mean germinability at time 0 is defined as the mean initial germinability (*g*_0_); in the freezing and light treatments, mean germinability at time 0 is defined as *g*_max_. The first intersection point of the mean germinability and *g*_max_/2 was used as an estimate for the half-life of the spores under the light treatment 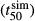. To clarify the different measures, *g*_0_, *g*_max_, *g*_max_/2, and 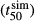 are marked in the panels for *P. punctatus* (D–F) with red labels; note that the former three measures are identical in all three panels.

We defined 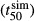 for each species as the time at which the mean germinability of the species in the light treatment, averaged over the different individuals, first dropped to 50% of the maximal mean germinability that was observed in the control treatment (*g*_max_; Fig. 2). As this time was typically not observed directly, we inferred it from the time series by assuming linear change in the log-transformed mean germinability between the observations. For this interpolation, germinability at time zero in the light treatment was assumed to equal *g*_max_.

Radiation at short UV wavelengths is generally more harmful to organisms than longer wavelengths. The relative biological effect of different wavelengths can be expressed by biological spectral weighing functions (BSWFs; e.g., McKenzie et al. 2011). We used the irradiance spectrum in the light treatment (Fig. 1A) and alternative BSWFs (Fig. 1B) to estimate the 50% viability-reducing biologically effective radiation dose for the spores of the different species. Further, we calculated the expected distance at which spores dispersing in direct sunlight (Fig. 1A) reach this viability level (henceforth called half-distance, *d*_50_) under different scenarios of mean wind speed and mean daily irradiance. Note that in the half-distance calculations, we are considering only those spores that remain airborne long enough to reach 50% viability level due to solar radiation. Thus, we are not making any assumption concerning the proportion of spores that this represents out of all spores released into the air, which depends on many factors. Generally, models of wind dispersal predict very long airborne times for particles in the 1–10 *μ*m size range (Wilkinson et al. 2012; Norros et al. 2014): for instance, (Wilkinson et al. 2012) estimated the expected airborne time of 9 *μ*m particles to be 2.2–10.4 days depending on the site of release.

We estimated the 50% viability-reducing biologically effective solar radiation dose (denoted LD_50_; unit mol m^−2^) for each species by calculating the biologically effective radiation dose the spores had received during their half-life 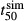 (units *s*). LD_50_ was calculated as LD_50_ = *I*_sim_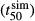, where *I*_sim_ is the biologically effective irradiance in the light treatment (unit mol m^−2^ sec^−1^), calculated as 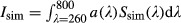, where *λ* is wavelength (unit nm), *S*_sim_ (*λ*) is the spectrum of the simulated sunlight (unit mol m^−2^ sec^−1^ nm^−1^), and *a* (*λ*) is the adopted BSWF (unitless). Similarly, the biologically effective irradiance in sunlight (unit mol m^−2^ sec^−1^) is 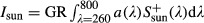 where GR is the global radiation (i.e., including both direct and reflected or scattered radiation; mol m^−2^ sec^−1^) at 260–4000 nm and 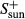 (unit nm^−1^) is the solar reference spectrum (adopted from Chu and Liu 2009) standardized by global radiation. Thus, the half-distance *d*_50_ (unit m) of spores dispersing under wind speed *u* (unit m sec^−1^) in direct sunlight (above the canopy or across an open area) can be calculated as *d*_50_ = 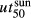, where 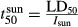 is the half-life of spores in sunlight.

We calculated *d*_50_ as a function of *u* for different values of 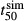 and GR for four alternative formulations for the weighing function *a* (*λ*). The first two were the BSWFs reported by Quaite et al. (1992) (as formulated by Musil 1995) for DNA damage in alfalfa (*Medicago sativa*) seedlings (W1, Fig. 1B) and by Flint and Caldwell (2003) for growth responses of oat (*Avena sativa*) seedlings (W2, Fig. 1B). The former strongly emphasizes the shortest wavelengths, while the latter gives considerable weight also to longer UV wavelengths; both have received some support in studies of UV damage on fungi (Paul et al. 2005). As these weighing functions apply to the effects of UV only and cannot reliably be extrapolated to longer wavelengths, we let *a* (*λ*) = 0 for *λ* > 400. In other words, we assumed that all mortality was caused by the UV and none by the visible part of the spectrum. The latter two BSWFs were simply based on the relative energy content per photon, applied for the UV region only (W3, Fig. 1B; *a* (*λ*) = 0 for *λ* > 400) or also for the visible and near-infrared regions (up to 800 nm) (W4, Fig. 1B).

During April–October, when most wood-decay fungi produce their spores (Nuss 1975), the minimum and maximum values of daily solar radiation (cloudy vs. sunny days) are roughly similar in each month, although the monthly mean values peak in the summer (Anonymous 1993). For GR, we used two different values (5 W m^−2^ and 320 W m^−2^ when converted from the quantum form to the more conventional energy units), chosen to represent the typical minimum and maximum daily values (averaged over a whole 24-h day) in Finland over April–October (Anonymous 1993). For 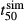, we used two different values, corresponding to the mean of the species-specific values for species with thick- and thin-walled spores, respectively.

### Modeling the effect of spore characteristics on viability

To statistically analyze the effect of the treatments and spore traits on spore viability, we adopted a hierarchical community modeling approach (HCM; Royle and Dorazio 2006; Ovaskainen and Soininen 2011). In other words, we modeled each response variable at the level of individual species and then combined the species-specific models into a single community-level model. The advantage of this approach is that we can analyze the effect of the light and freezing treatments for each individual species as well as the influence of species traits on each treatment response in a single model fitting. In this approach, data from all species inform the community-level parameters, which in turn affect the predictions obtained for each individual species. Apart from quantifying the trait effects, the community-level parameters give a useful summary of the response of an average species in the community.

We examined whether species-specific spore traits explain three response variables: initial germinability *g*_0_, light resistance *R*_L_, and freezing resistance *R*_F_. Spore volume (log-transformed; log *V*), residual spore elongation (*e′*), and spore wall thickness (*w*) were used as covariates (Table 1). Residual spore elongation *e′* was adopted instead of spore elongation *e*, as the latter does not vary independently of *w* among the study species but is larger for thin-walled (mean: 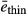 = 2.4) than for thick-walled spores (mean: 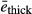 = 1.2; *t*-test: *P* = 2 × 10^−4^). We defined residual spore elongation as *e′* = *e* – 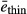 or *e′* = *e* – 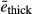 for thin- or thick-walled species, respectively. All covariates were standardized to have zero mean and unit variance.

**Table 1:** The study species, the traits of their spores, and responses to the treatments. For the predicted light and freezing resistance, we show the posterior median value as well as the posterior probability (%) that the response is negative (denoted *P*_post_ (*γ*_*i*_ < 0); calculated based on a sample of 10,000 values from the posterior distribution). The spore trait values are based on Niemelä (2005)

	Species traits			Responses to treatments				
Variable/parameter	Spore volume (*μ*m^3^, log-transformed)	Spore elongation (mean length/mean width)	Spore wall thickness (thin (0)/thick (1))	Spore half-life in light treatment (h) (see Estimating the effect of light-induced reduction in germinability on dispersal distances)	Predicted light resistance as estimated by the HCM		Predicted freezing resistance as estimated by the HCM	
Symbol	log *V*	*e*	*w*	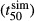	*γ*_*i*_		*γ*_*i*_	
Species					Posterior median	*P*_post_ (*γ*_*i*_ < 0)	Posterior median	*P*_post_ (*γ*_*i*_ < 0)
*Antrodia serialis*	3.4	2.5	0	1.2	−2.37	100.0	−0.57	99.8
*Antrodiella pallescens*	1.9	1.8	0	1.7	−1.71	100.0	−0.57	99.9
*Bjerkandera adusta*	2.7	1.7	0	2.1	−1.28	100.0	−0.63	100.0
*Cerrena unicolor*	3.0	1.7	0	1.7	−2.04	100.0	−1.23	100.0
*Datronia mollis*	3.9	2.8	0	1.2	−2.40	100.0	−0.57	99.8
*Fomes fomentarius*	5.6	3.3	0^1^	1.7	−1.86	100.0	−0.16	78.7
*Fomitopsis pinicola*	4.1	1.9	0	1.4	−1.92	100.0	−0.69	100.0
*Hapalopilus rutilans*	2.3	1.6	0	1.2	−1.79	100.0	−0.55	99.7
*Inonotus radiatus*	3.5	1.4	1	3.5	−0.85	99.4	−0.42	98.5
*Phellinus igniarius s.l*.	4.5	1.2	1	22.6	−0.29	81.1	−0.85	100.0
*Phellinus laevigatus*	3.5	1.3	1	1.9	−1.21	100.0	−0.53	99.5
*Phellinus punctatus*	4.7	1.1	1	19.4	−0.40	88.6	−0.74	100.0
*Phellinus viticola*	2.4	3.7	0	1.5	−1.79	100.0	0.02	45.4
*Postia tephroleuca*	1.6	3.3	0	1.5	−2.61	100.0	−0.02	54.1
*Rigidoporus populinus*	3.2	1.1	1	3.4	−0.66	97.3	−0.50	99.6
*Skeletocutis amorpha*	1.1	2.7	0	1.6	−1.28	100.0	−0.09	67.1
*Trichaptum abietinum*	3.1	2.3	0	0.7	−4.08	100.0	−0.35	96.5

1 The classification of *F. fomentarius* spore walls was less straightforward, as Niemelä (2005) describes them as “thickish”; however, we considered them to be closer to those of thin-walled rather than those of truly thick-walled spores.

We modeled each response variable separately, thus resulting in three univariate models. In one univariate model, let *y*_*ij*_ denote the log- or logit-transformed response variable for an individual *j* of species *i*. We used logit transformation for initial germinability (*g*_0_) (which has values between 0 and 1) and log transformation for light (*R*_L_) and freezing resistance (*R*_F_) (which can also have values >1). Because the observed values of all response variables included zeroes, we added a small number (0.01) to all observed values before transformation. We modeled the transformed response variable as


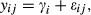
(1)

where *γ*_*i*_ is the predicted value of the response variable for species *i* and the residual *ε*_*ij*_ is assumed to follow a normal distribution with zero mean and variance *τ*^2^, describing the unexplained variance among individuals within species *i*.

The predicted species-specific response *γ*_*i*_ was related to spore traits (*V*, *w* and *e*') according to the following equation:


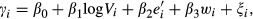
(2)

where the residual *ξ*_*i*_ is normally distributed with zero mean and variance *ψ*^2^, describing the variance among species that is not explained by the spore traits.

We estimated probability distributions for the values of parameters (*γ*_*i*_, *τ*^2^, *β*_0_*–β*_3_, *ψ*^2^) by a Bayesian approach (Gelman et al. 2004). We assumed a Gaussian prior distribution with zero mean and standard deviation of 10 (N(0, 10^2^)) for the regression coefficients *β*_0_*–β*_3_. For the variance parameters *ψ*^2^ and *τ*^2^, we assumed an inverse Wishart prior distribution (Inv-Wishart _*t*0_(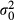); Gelman et al. 2004) with *t*_0_ = 3 degrees of freedom and the one-dimensional identity matrix as the scale matrix 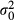. As we used conjugate priors for all parameters, the posterior probability distributions for the parameters could be Gibbs sampled directly from the conditional posterior distributions (Gelman et al. 2004). For details of the sampling, see Supplementary material Appendix S1.

For each species, the effect of the treatments was evaluated by computing the posterior probability that the predicted light or freezing resistance (*γ*_*i*_) is negative, corresponding to a negative response to the treatment. Further, for the response variables *R*_L_ and *R*_F_, the community-level parameter *β*_0_ describes the mean level of light or freezing resistance over all species, a negative value indicating a negative overall response in the studied species group. Note that, by contrast, for the logit-transformed *g*_0_, a negative (positive) *β*_0_ means that the mean level of initial germinability is below (above) 50%. The effect of the spore traits on *g*_0_, *R*_L_, and *R*_F_ was evaluated as the posterior probability that the corresponding coefficients *β*_1_*–β*_3_ are negative or positive. The estimated model parameter values presented in Tables 1 and 2 correspond to the transformed and standardized variables. In the figures, all variables are shown in their untransformed and nonstandardized form unless otherwise mentioned.

**Table 2:** The estimated posterior median values for regression and variance parameters at the community level. For the parameters describing the effect of treatments and spore traits, we show also the posterior probability (%) that the effect is positive or negative (denoted *P*_post_ (*β*_*x*_ ≠ 0); calculated based on a sample of 10,000 values from the posterior distribution), corresponding to the sign of the median. See equations (1–2) in Modelling the effect of spore characteristics on viability for the model equations

		Response variable					
		Initial germinability (*g*_0_)		Light resistance (*R*_L_)		Freezing resistance (*R*_F_)	
Parameter in HCM	Represented effect	Posterior median	*P*_post_ (*β*_*x*_ ≠ 0)	Posterior median	*P*_post_ (*β*_*x*_ ≠ 0)	Posterior median	*P*_post_ (*β*_*x*_ ≠ 0)
*β*_0_	Mean level	−0.59		−1.68	100.0	−0.50	100.0
*β*_1_	Spore volume (Log *V*)	0.72	99.8	−0.05	58.4	−0.11	83.3
*β*_2_	Residual spore elongation (*e*′)	0.16	78.8	−0.08	64.2	0.24	98.7
*β*_3_	Spore wall thickness (*w*)	−0.92	100.0	0.68	99.7	−0.04	62.7
*τ*^2^		0.29		0.66		0.24	
*ψ*^2^		0.60		0.54		0.11	

## Results

Initial germinability *g*_0_ varied considerably among the 17 examined species, ranging from 10-20% (e.g., most species with thick-walled spores) to over 90% (*Fomes fomentarius*) (Supplementary material Appendix S2, Fig. S1). Across species, the mean level of germinability was 36% (median *β*_0_ = −0.59 ≈ logit (0.36) for *g*_0_; Table 2). Generally, there were no drastic changes in the average germination rate in the course of the 48 h of control treatment. In most species, germinability either decreased or peaked at intermediate hours.

Both light and freezing treatments had a negative effect on the germinability of most species (Table 1; Fig. 2; Supplementary material Appendix S2, Fig. S1). The negative effect of light was statistically supported by >95% posterior probability in all but two cases (*Phellinus igniarius* and *P. punctatus*, Table 1). For many species, the effect of light was quite dramatic, decreasing the germinability to less than 20% of the maximum during the first 4 h. The effect of freezing was generally smaller and decreased germinability for 13 of the 17 species (supported by > 95% posterior probability; Table 1). The mean levels of light (*R*_L_) and freezing resistance (*R*_F_) across species (*β*_0_, eq. 2) were negative with 100.0% posterior probability, with a median of −1.68 and −0.50, respectively (Table 2). Thus, the negative effect of both light and freezing was strongly supported also at the community level.

**Figure 3:**
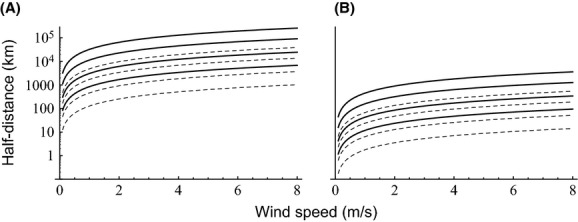
The distance at which the germinability of dispersing spores is expected to drop by 50% due to exposure to sunlight, as a function of average wind speed. Panels (A; 5 Wm^−2^ and B; 320 Wm^−2^) correspond to typical minimum and maximum amounts of daily radiation (i.e., cloudy vs. sunny days) for the species examined, as measured in Finland over April–October in open areas (Anonymous 1993). Solid lines: thick-walled spores; dashed lines: thin-walled spores. The four lines correspond to the different spectral weighing functions W1–W4 (from highest to lowest line) described in Estimating the effect of light-induced reduction in germinability on dispersal distances.

The half-lives of spores in the light treatment ranged from 40 min (*Trichaptum abietinum*) to over 20 h (*P. igniarius*) (Table 1). The mean half-life was 1.5 h for thin-walled species and 10 h for thick-walled species. Correspondingly, the half-distances calculated based on the half-lives and the wavelength spectra of simulated and real sunlight were an order of magnitude lower for thin-walled species (Fig. 3). However, the irradiance conditions were even more critical for the predicted dispersal mortality than spore type, with half-distances changing by two orders of magnitude from the darkest to lightest days as measured during the Finnish growth season (Anonymous 1993). According to our calculation, on the darkest days, light-induced mortality is likely to restrict dispersal only at the largest spatial scales (hundreds–thousands of kilometers). On the brightest days, light-induced mortality is important at shorter distances, but the precise spatial scale could not be identified based on our data, as it is sensitive to the assumed BSWFs, that is, to the relative roles of different wavelengths in causing the light damage observed in the laboratory treatments.

**Figure 4:**
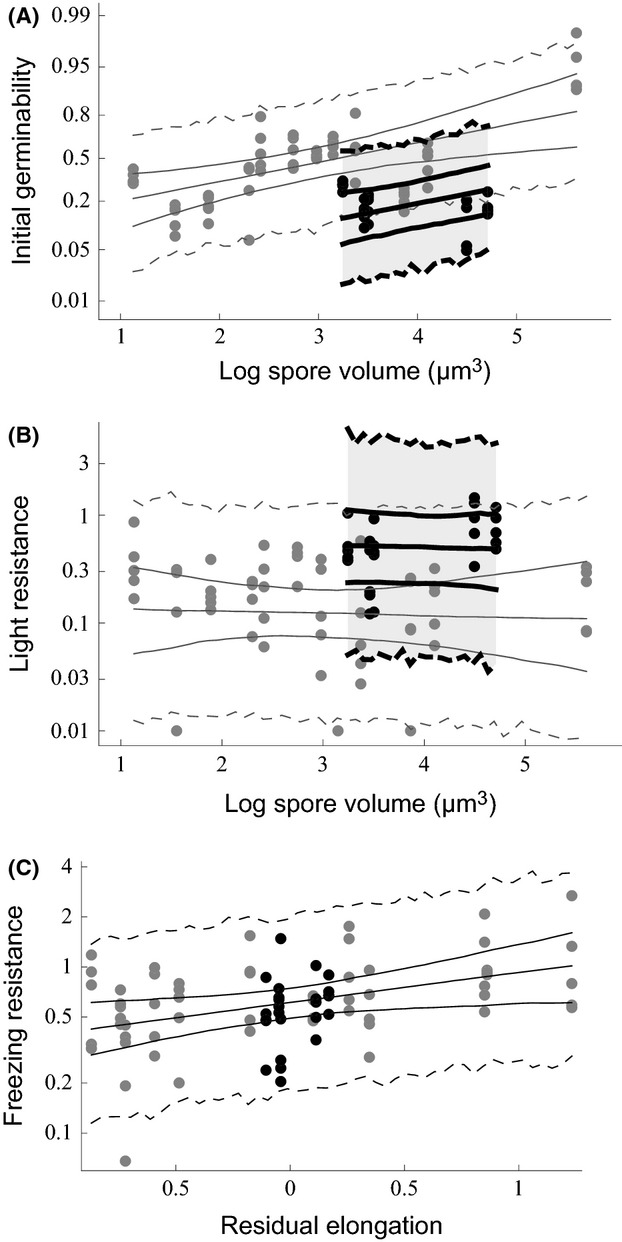
The effects of spore traits on initial germinability (A) and light (B) and freezing resistance (C) of spores. Each point corresponds to spores from a fruit body growing on a separate trunk, that is, to a fungal individual. The lines show the median and 95% credibility intervals of the HCM, including parameter uncertainty only (solid lines) or also random variation among species and individuals (dashed lines). Black points and thick black lines: species with thick-walled spores; gray points and lines: species with thin-walled spores. In the plotted model predictions, those spore traits whose effect is not illustrated were always assumed to have the mean trait value over all species.

Species-specific freezing (*R*_F_) and light resistance (*R*_L_) and initial germinability (*g*_0_) depended on spore traits (Table 2, Fig. 4). Initial germinability *g*_0_ was higher for species with larger spores (positive *β*_1_ for *g*_0_). Species with thick-walled spores had lower initial germinability (negative *β*_3_ for *g*_0_) but higher resistance to the light treatment (positive *β*_3_ for *R*_L_). Less intuitively, species with more elongated spores (after accounting for the correlation between elongation and spore wall thickness) were more resistant to the freezing treatment (positive *β*_2_ for *R*_F_). The median unexplained variance between individuals of the same species (*τ*^2^) ranged from 0.24 to 0.66 and the median among-species variance (*ψ*^2^) from 0.11 to 0.60 depending on the response variable (Table 2). Neither type of unexplained variance was consistently larger than the other.

## Discussion

In this study, we examined the effects of external conditions and species-specific spore traits on the germination of the spores of wood-decay fungi, with the aim of improving the understanding of the costs of dispersal in this group of species. The germinability of the spores of our study species was clearly sensitive to the freezing and especially to the light treatment. Species with thin-walled spores were particularly sensitive. This result is in good accordance with several earlier studies that have demonstrated a harmful effect of light on the spores of fungal species belonging to other groups (Buller 1909; Gregory 1973; Rotem and Aust 1991; Parnell et al. 1998; Mitakakis et al. 2003; Isard et al. 2006; Kanetis et al. 2010). Few others have examined the effect of species traits on light sensitivity, although Rotem and Aust (1991) reported differences in the sensitivity of different species and Gregory (1973) mentioned that pigmented spores appear to be more resistant. Our study extends the knowledge of harmful light effects to wood-decay fungi, confirms the species-specific responses to harmful light, and relates them to spore traits such as size and wall properties.

In most previously studied species, the rate of survival reduction was slower than among our study species (especially those with thin-walled spores). This could be partly due to methodological differences, e.g., in the quality of the light, which has in most cases not been reported in detail. It seems also feasible that the forest-dwelling wood-decay fungi are generally more sensitive to light than the plant pathogens and molds that form the majority of the previously studied species, as the latter are typically not restricted to shady forest conditions. Low survival under exposure to light may be typical for basidiospores: Kramer and Pady (1968) found that the viability of airborne basidiospores is generally low and often lower for spores sampled from the air during the day, while such a diurnal pattern in viability is absent in other fungal spore types. As a possible adaptation to loss of viability under light, saprotrophic basidiomycetes generally release the majority of their spores during the night (Kramer 1982).

While it is clear that light is harmful for the small spores of wood-decay fungi, the spatial scale at which light-induced dispersal mortality can be expected to be relevant is a more complex issue. The sensitivity of the calculated half-distances to the assumed BSWF shape (Figs. 1 and 4) shows that the critical spatial scale depends on the relative effect of different wavelengths, that is, on the specific mechanism responsible for the reduced viability. Unfortunately, the BSWFs that we used were based on measurements with other organisms, and without careful empirical measurements, there is no obvious way to decide which might be the closest to the truth in the case of radiation damage to the spores of wood-decay fungi. As there was more shorter-wavelength UV (260–300 nm) in our treatments than under typical solar irradiance conditions, mortality in nature could be considerably lower if most of the damage was caused by the short wavelengths. However, an effect of real sunlight on fungal spores has been reported at least by Buller (1909), Rotem and Aust (1991), and Kanetis et al. (2010), supporting the relevance of light as a mortality factor in nature. Buller (1909) even observed decreased germination rate for spores treated with sunlight coming through an ordinary glass window, which should cut off wavelengths below 315 nm and reduce intensity even at longer UV wavelengths.

The extrapolation of laboratory responses to nature is further complicated by possible interactions between sunlight and other physical factors in the atmosphere. There is some evidence that low temperature and dry conditions in the atmosphere may protect airborne propagules from radiation damage (Gregory 1973; Rotem et al. 1985). By contrast, Peccia et al. (2001) found that high relative humidity ameliorated the negative effect of UV on airborne bacteria. Furthermore, as pointed out in the Material and Methods (Treatments and culturing), some of the decrease in germination could represent delayed germination instead of true mortality, although our subsequent empirical tests do not support this (J. Nordén, E. Karhu, O. Ovaskainen, A. V. Vähätalo, K.-H. Larsson, M. Edman, V. Norros, unpubl. ms.). Acknowledging this possibility is important, as delaying germination after experiencing adverse conditions could in fact be adaptive. On the other hand, the risk of predation by invertebrates and being overgrown by faster competitors could counter any benefits of waiting for improved conditions, especially given the intense competition between wood-decay fungi for the limited resources within a substrate unit (Woodward and Boddy 2008). In any case, it is clear that measurements under more natural conditions are ultimately needed to confirm and quantify the relevance of the mortality factors we have uncovered in this pioneering study in nature. However, designing such a study is not straightforward due to the various challenges in detecting, obtaining, and handling the microscopic spores, and in avoiding contamination of the spore samples.

While our results are inconclusive as to whether light-induced mortality can limit the dispersal of fungi between individual forest sites within a landscape, our experiments certainly show that light-induced loss in germinability can be an important factor restricting the airborne lifetime of species with thin-walled spores. Models of airborne dispersal show that the rate of long-distance dispersal depends strongly on the longevity of propagules in the atmosphere (Aylor 2003; Wilkinson et al. 2012). In the long run, this could have important implications, for example, for the rate of population spread and gene flow across long distances (Trakhtenbrot et al. 2005). For plant species, Soons and Ozinga (2005) found that species-specific differences in the probability of rare long-distance dispersal events were more critical for the species' regional survival probability in a fragmented landscape than differences in median dispersal distances.

Although our experiments did not simulate the specific conditions on woody substrates at the forest floor, it is conceivable that solar radiation and extreme temperatures could cause mortality also at the settlement phase. Very little is known about the occurrence of secondary dispersal of landed spores and about the establishment process in wood-decay fungi. However, resuspension of microscopic particles back into the air is generally low under moist conditions such as those typical of the boreal forest floor (Hinds 1999), so it can be expected that the majority of spores stay approximately where they land. Moreover, many species of wood-decay fungi (including those studied here) are considered unit-restricted, which means that they can only establish and grow directly on the woody substrate (Boddy and Heilmann-Clausen 2008). Thus, establishment is likely to be a critical transition in the life cycle of these species, and the rare windows of colonization opportunity could be made still narrower by sensitivity to physical conditions. Freezing could be an important mortality factor especially for species that produce spores late in the autumn and thus risk not being able to colonize the substrate before the onset of winter. Decreased establishment rates due to a more adverse microclimate could be one reason behind the lower probability of species occurrence at habitat edges (Snäll and Jonsson 2001; Siitonen et al. 2005). Further, the sensitivity to external conditions and readiness to germinate without specific cues would suggest that at least those wood-decay fungi with thin-walled spores are unlikely to form a long-lived spore bank. This would be in contrast to the ectomycorrhizal *Rhizopogon* species studied by Bruns et al. (2009) which retained and even increased their ability to infect tree roots after four years in the soil. In wood-decay fungi, a spore bank could in principle form either directly on dead wood, on living wood waiting for the tree to die, or in the soil waiting for a piece of wood to land – with different external conditions in each case. As the existence of a spore bank would have very important consequence to the species' dynamics, it would be interesting to confirm the decrease of the viability of settled spores under natural conditions by an experimental approach comparable to that of Bruns et al. (2009).

Our results have interesting implications for the different evolutionary pressures that control and constrain spore traits in fungi. The positive relationship between spore size and germination rate probably reflects the higher amount of resources available for germination in larger spores. As larger spores have a higher deposition rate (Petroff et al. 2008; Hussein et al. 2013) and thus a generally shorter expected airborne lifetime (Norros et al. 2014), this result indicates a trade-off between dispersal distance and establishment probability in wood-decay fungi. Although such a relationship has often been demonstrated for plant seeds (e.g., Skarpaas et al. 2011), to our knowledge, this is the first study confirming it for a fungal group. The lower initial germinability (*g*_0_) of thick-walled spores suggests another trade-off between spore light resistance and establishment rate: Thick walls enhance spore survival at the cost of germinability or germination speed, potentially giving a competitive advantage to thin-walled spores in the critical initial stages of establishment on new substrate. By contrast, the higher freezing resistance (*R*_F_) of more elongated spores does not immediately suggest any simple biological explanation, and we leave it to be confirmed by future work.

It would be interesting to extend the discussion of the correlations between germinability and resistance and other spore traits also to further species traits of wood-decay fungi. For instance, species that release spores in the late spring and summer face more intense radiation but a lower risk of freezing than those fruiting in the late autumn. Thus, one could hypothesize that spring-producing species have higher light resistance and autumn-producing species higher freezing resistance than other species. Unfortunately, spore-producing season and other central life-history traits such as fecundity are still largely unstudied for wood-decay species. Among our study species, there was no obvious pattern in the light or freezing resistance of species sampled (that were thus actively producing spores) during the spring vs. during the autumn, other than the fact that the two species (*Phellinus igniarius* and *P. punctatus*) whose spores had much longer half-lives under the light treatment than other species were among the spring producers.

Alongside wood-decay fungi, organisms from other taxonomic groups have to cope with harmful UV radiation. The UV-sensitive parts of cells such as DNA in nucleus or enzymes in cytoplasm can be protected by UV-absorbing sunscreens (e.g., mycosporine-like amino acids) in the outer parts of cells (e.g., cell wall; Piiparinen et al. 2015). Sunscreens can protect large (>10 *μ*m) organisms effectively, but provide no protection for smallest (<1 *μ*m) organisms (e.g., bacteria and viruses; Garcia-Pichel 1994). Organism like fungal spores between 1 *μ*m and 10 *μ*m in size requires large investments for sunscreens (like thick cell walls in this study or high concentrations of sunscreens) to receive effective protection against UV radiation (Garcia-Pichel 1994). Our results show that the thick cell wall of some species provides some protection, but at the cost of reduced overall germinability within 24 h. Another possible interpretation is that thick-walled spores are resting stages that require a longer time for germination and not only the cell wall but also the inactive state as such protects the spores against UV damage. Similarly, the inactive resting spores of many bacteria are more resistant to UV radiation than the active cells (Nicholson et al. 2000).

We conclude that in wood-decay fungi, freezing and especially sunlight are likely to be important sources of dispersal mortality at both transfer and settlement phases. Thus, from the point of view of wood-decay fungi, the connectivity of a landscape may be affected by the openness of the habitats that make up the matrix, as well as the weather conditions during the dispersal phase. These costs of dispersal can make the colonization of isolated habitat patches and habitat edges difficult, and more difficult for some species than others, depending on species traits. Different costs of dispersal could be one of the reasons for the high variation in wood-decay species' responses to habitat fragmentation (Nordén et al. 2013).

## Supporting Information

**Appendix S1.** Sampling the posterior distributions of the Hierarchical Community Model parameters.

**Appendix S2.** Plots of the species-specific responses to the light and freezing treatments.

**Figure S1.** The change in germinability over time (h) in the control (dark at 25°C), light (simulated sunlight at 25°C) and freezing (dark at −25°C) treatments.
