# Supplementary material for: Spore sensitivity to sunlight and freezing can restrict dispersal in wood-decay fungi
Source: Ecol Evol. 2015 Jul 22;5(16):3312–26. doi: 10.1002/ece3.1589 (PMC4569028; doi:10.1002/ece3.1589)
Supplement: Supplementary file 1 — Appendix S1. Sampling the posterior distributions of the Hierarchical Community Model parameters. [file ece30005-3312-sd1.docx]

Supporting information

Appendix 1

Sampling the posterior distributions of the Hierarchical Community Model parameters

Below, we describe how we sampled the posterior distributions of the parameters of the hierarchical community model (HCM). For definitions of the variables, see the main text. For the sake of clarity, the main text describes the HCM in a scalar form. However, in the sampling, most parameters existed as vectors or their combinations (matrices), shown in bold below. The notation ***I_n_*** indicates an *n*-by-*n* identity matrix, where *n* is a positive integer.

For the sampling, we combined the species-specific parameters γ*_i_* into a single vector ***γ*** = (*γ*_1_, … , *γ*_m_), where *m* = 17 is the number of study species. The linear regression model at the community level was:

**γ** = ***Xβ****+****δ*** ,

where ***β*** is a vector combining the *h* = 4 regression parameters *β*_0_*-β*_3_, ***X*** is the *m* x *h* design matrix that contains the values of the spore covariates, and ***δ*** ~ N(**0**, ***W***), where ***W*** = *ψ*^2^***I_m_***, *ψ*^2^ is the variance among species that is not explained by the spore traits.

Each γ*_i_* was sampled from

γ*_i_* | ***β***, *ψ*^2^, τ^2^, *y_ij_* ~  N(*b_i_*, *u_i_*^2^),

where *u_i_*^2^ = (*n_i_* τ^-2^ + *ψ*^-2^)^-1^, *b_i_* = $u_{i}^{2}\left(\frac{1}{\psi^{2}}\left(X\beta\right)_{i}+\frac{1}{\tau^{2}}\sum_{j=1}^{n_{i}} y_{ij}\right)$ and *n_i_* is the number of sampled individuals of species *i*.

*τ*^2^ was sampled from

*τ*^2^ | γ*_i_*, *y_i_* ~  Inv-Wishart *_t_*$\left(\sigma_{0}^{2}+\left(\sum_{i=1}^{m} \sum_{j=1}^{n_{i}} \left(c_{ij}-\bar{c}_{i}\right)^{2}\right)I{}_{1}\right)$,

where *t* = *t*_0_ + *N*, $c_{ij}=y_{ij}-\gamma_{i}$, $\bar{c}=\frac{1}{N}\sum_{i=1}^{m} \sum_{j=1}^{n_{i}} c_{ij}$ , and $N=\sum_{i=1}^{m} n_{i}$, i.e. the total number of sampled individuals. *t*_0_ is the degrees of freedom parameter of the Inv-Wishart distribution (we used *t*_0_ = 3) and *σ_0_*^2^ is the assumed scale matrix, here the one-dimensional identity matrix (i.e. *σ_0_*^2^ = 1).

***β*** was sampled from

***β***|***γ***, *ψ*^2^ ~ N(***b***, ***R***),

where ***R*^-1^** = ***X* ^T^*W*^-1^*X*** + **Z^-1^** and ***b*** = ***RX* ^T^*W*^-1^*γ***. ***Z*** = 10^2^***I_h_*** is the variance-covariance matrix of the multinormal prior distribution assumed for ***β***.

Finally, *ψ*^2^ was sampled from

*ψ*^2^ | ***β***, ***γ*** ~  Inv-Wishart *_v_*$\left(\sigma_{0}^{2}+\left(\sum_{i=1}^{m} \left(z_{i}-\bar{z}\right)^{2}\right)I{}_{1}\right)$,

where *v* = *t*_0_ + *m*, *z_i_* = γ*_i_* – (***Xβ***)*_i_* , and $\bar{z}=\frac{1}{m}\sum_{i=1}^{m} z_{i}$.
