# Supplementary material for: Spore sensitivity to sunlight and freezing can restrict dispersal in wood-decay fungi
Source: Ecol Evol. 2015 Jul 22;5(16):3312–26. doi: 10.1002/ece3.1589 (PMC4569028; doi:10.1002/ece3.1589)
Supplement: Supplementary file 2 — Appendix S2. Plots of the species-specific responses to the light and freezing treatments. [file ece30005-3312-sd2.docx]

Supporting information

Appendix 2

Plots of the species-specific responses to the light and freezing treatments

Figure A1. The change in germinability over time (h) in the control (dark at 25°C), light (simulated sunlight at 25°C) and freezing (dark at -25°C) treatments. The grey points connected by each thin grey line show the germinability of spores from one sampled individual in the course of the treatment, with the value at time 0 showing the initial germinability *g*_0_ (i.e. germinability without treatment). The thick black line is the mean germinability in each treatment, averaged over the different individuals. The horizontal dotted and dashed lines show respectively the maximal (*g_max_*) and 50% of the maximal (*g_max_*/2) mean germinability observed in the control treatment. In the control treatment, mean germinability at time 0 is defined as the mean initial germinability (*g*_0_); in the freezing and light treatments, mean germinability at time 0 is defined as *g_max_*. The first intersection point of the mean germinability and *g_max_*/2 was used as an estimate for the half-life of the spores under the light treatment ($\text{t}_{\text{50}}^{\text{sim}}$).
