# Supplementary material for: Spore sensitivity to sunlight and freezing can restrict dispersal in wood-decay fungi
Source: Ecol Evol. 2015 Jul 22;5(16):3312–26. doi: 10.1002/ece3.1589 (PMC4569028; doi:10.1002/ece3.1589)
Supplement: Supplementary file 3 — Figure S1. The change in germinability over time (h) in the control (dark at 25°C), light (simulated sunlight at 25°C) and freezing (dark at −25°C) treatments. [file ece30005-3312-sd3.pdf]

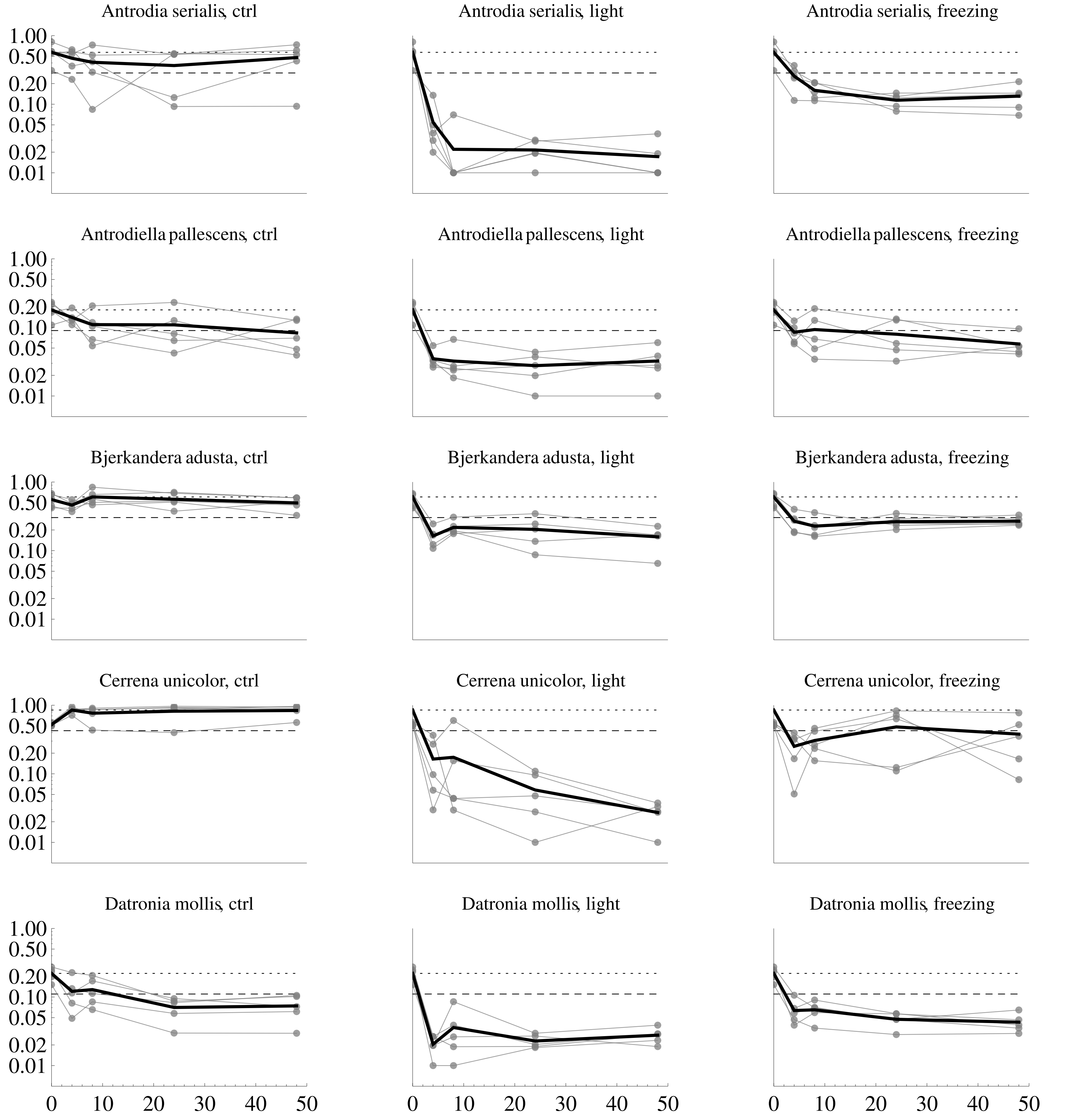

Antrodia serialis, ctrl
Antrodia serialis, light
Antrodia serialis, freezing
Antrodiella pallescens, ctrl
Antrodiella pallescens, light
Antrodiella pallescens, freezing
Bjerkandera adusta, ctrl
Bjerkandera adusta, light
Bjerkandera adusta, freezing
Cerrena unicolor, ctrl
Cerrena unicolor, light
Cerrena unicolor, freezing
Datronia mollis, ctrl
Datronia mollis, light
Datronia mollis, freezing
1.00
0.50
0.20
0.10
0.05
0.02
0.01
0
10
20
30
40
50

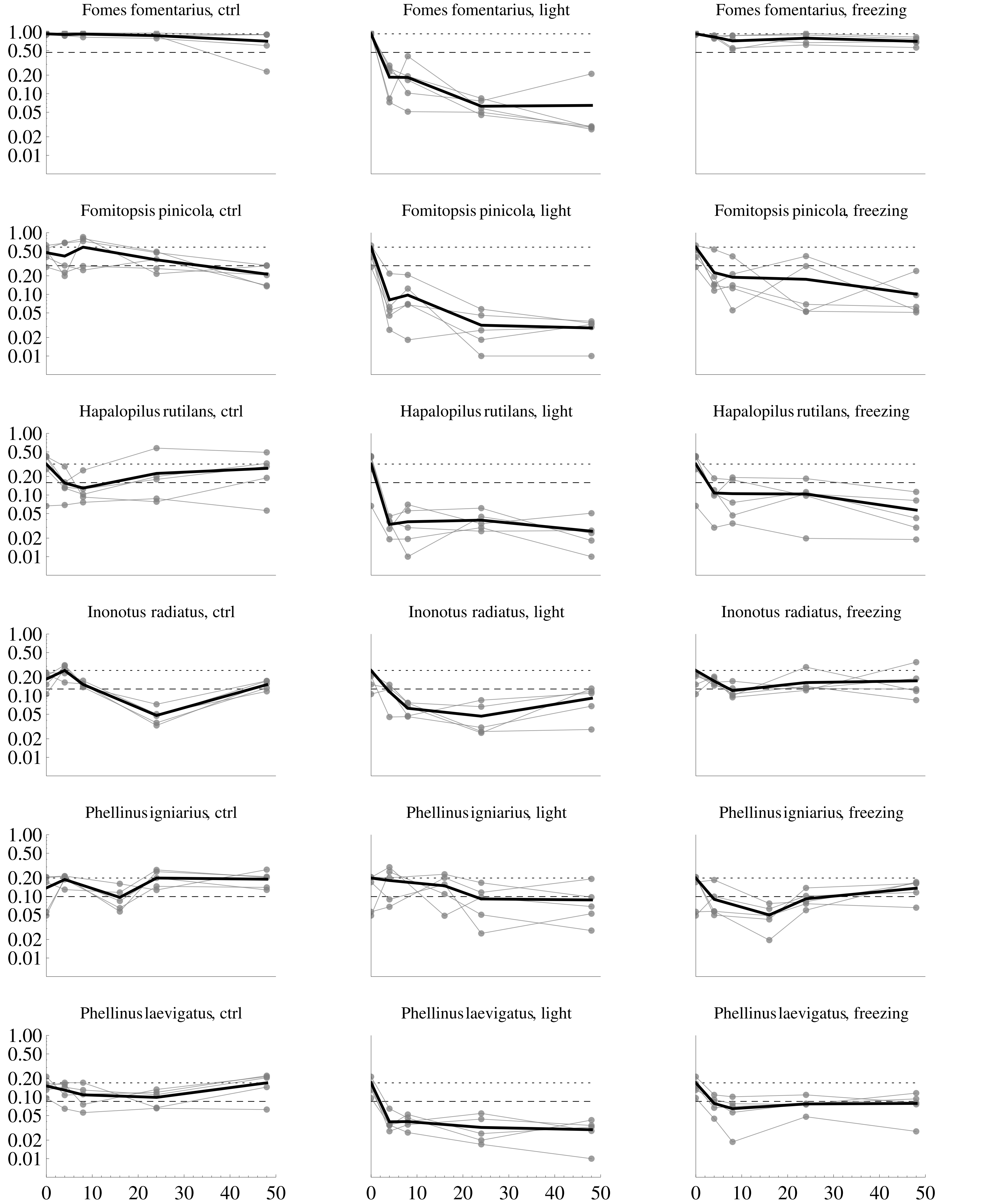

Fomes fomentarius, ctrl
Fomes fomentarius, light
Fomes fomentarius, freezing
Fomitopsis pinicola, ctrl
Fomitopsis pinicola, light
Fomitopsis pinicola, freezing
Hapalopilus rutilans, ctrl
Hapalopilus rutilans, light
Hapalopilus rutilans, freezing
Inonotus radiatus, ctrl
Inonotus radiatus, light
Inonotus radiatus, freezing
Phellinus igniarius, ctrl
Phellinus igniarius, light
Phellinus igniarius, freezing
Phellinus laevigatus, ctrl
Phellinus laevigatus, light
Phellinus laevigatus, freezing
1.00
0.50
0.20
0.10
0.05
0.02
0.01
0
10
20
30
40
50

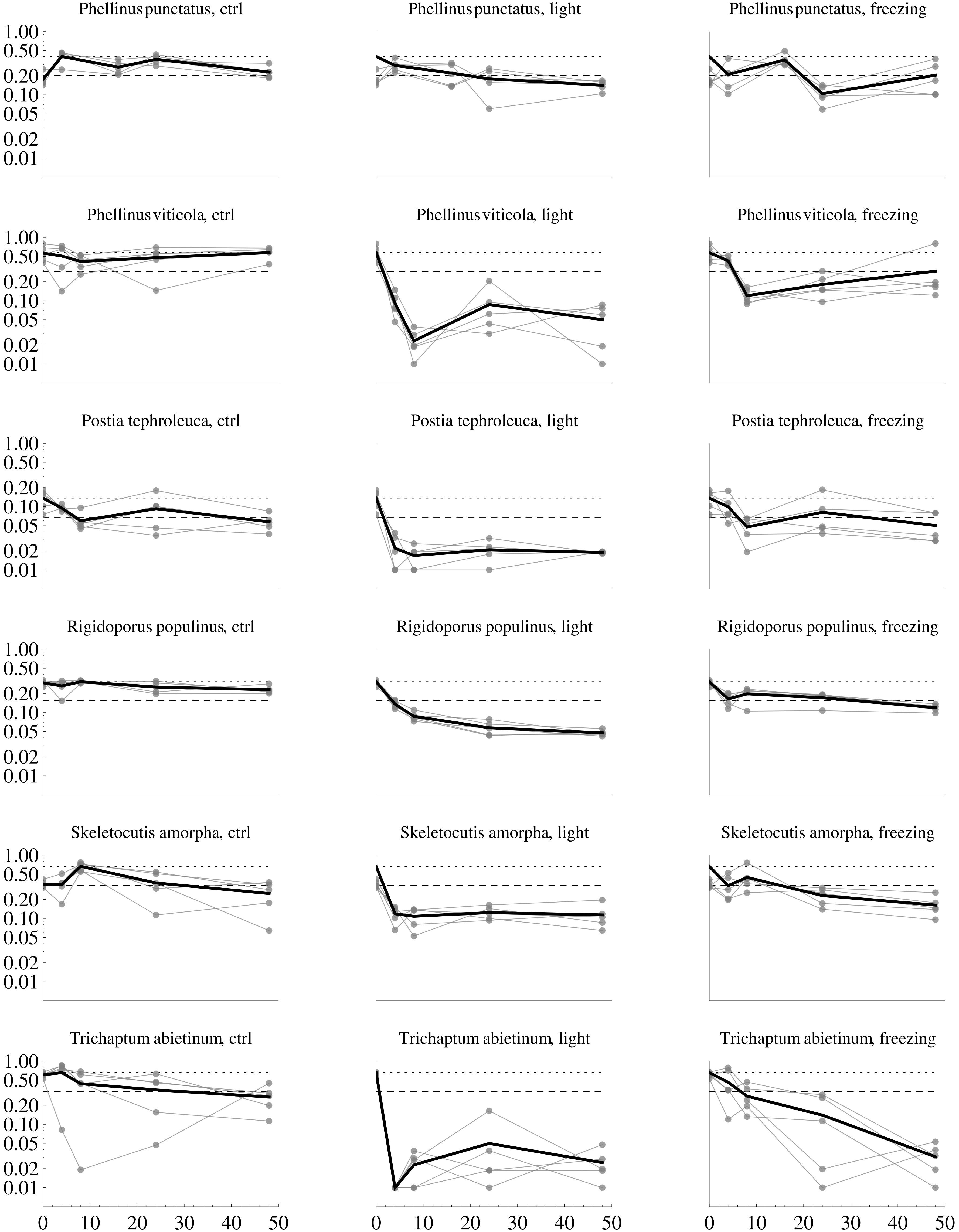
Phellinus punctatus, ctrl
Phellinus punctatus, light
Phellinus punctatus, freezing
Phellinus viticola, ctrl
Phellinus viticola, light
Phellinus viticola, freezing
Postia tephroleuca, ctrl
Postia tephroleuca, light
Postia tephroleuca, freezing
Rigidoporus populinus, ctrl
Rigidoporus populinus, light
Rigidoporus populinus, freezing
Skeletocutis amorpha, ctrl
Skeletocutis amorpha, light
Skeletocutis amorpha, freezing
Trichaptum abietinum, ctrl
Trichaptum abietinum, light
Trichaptum abietinum, freezing
1.00
0.50
0.20
0.10
0.05
0.02
0.01
0
10
20
30
40
50
